# Little Cross-Feeding of the Mycorrhizal Networks Shared Between C_3_-*Panicum bisulcatum* and C_4_-*Panicum maximum* Under Different Temperature Regimes

**DOI:** 10.3389/fpls.2018.00449

**Published:** 2018-04-06

**Authors:** Veronika Řezáčová, Lenka Zemková, Olena Beskid, David Püschel, Tereza Konvalinková, Martina Hujslová, Renata Slavíková, Jan Jansa

**Affiliations:** Laboratory of Fungal Biology, Ecology, Institute of Microbiology, Czech Academy of Sciences, Prague, Czechia

**Keywords:** C_3_ and C_4_ photosynthesis, common mycorrhizal networks (CMNs), temperature, natural ^13^C isotopic abundance, *Panicum* sp., arbuscular mycorrhiza, community, quantitative real-time PCR

## Abstract

Common mycorrhizal networks (CMNs) formed by arbuscular mycorrhizal fungi (AMF) interconnect plants of the same and/or different species, redistributing nutrients and draining carbon (C) from the different plant partners at different rates. Here, we conducted a plant co-existence (intercropping) experiment testing the role of AMF in resource sharing and exploitation by simplified plant communities composed of two congeneric grass species (*Panicum* spp.) with different photosynthetic metabolism types (C_3_ or C_4_). The grasses had spatially separated rooting zones, conjoined through a root-free (but AMF-accessible) zone added with ^15^N-labeled plant (clover) residues. The plants were grown under two different temperature regimes: high temperature (36/32°C day/night) or ambient temperature (25/21°C day/night) applied over 49 days after an initial period of 26 days at ambient temperature. We made use of the distinct C-isotopic composition of the two plant species sharing the same CMN (composed of a synthetic AMF community of five fungal genera) to estimate if the CMN was or was not fed preferentially under the specific environmental conditions by one or the other plant species. Using the C-isotopic composition of AMF-specific fatty acid (C16:1ω5) in roots and in the potting substrate harboring the extraradical AMF hyphae, we found that the C_3_-*Panicum* continued feeding the CMN at both temperatures with a significant and invariable share of C resources. This was surprising because the growth of the C_3_ plants was more susceptible to high temperature than that of the C_4_ plants and the C_3_-*Panicum* alone suppressed abundance of the AMF (particularly *Funneliformis* sp.) in its roots due to the elevated temperature. Moreover, elevated temperature induced a shift in competition for nitrogen between the two plant species in favor of the C_4_-*Panicum*, as demonstrated by significantly lower ^15^N yields of the C_3_-*Panicum* but higher ^15^N yields of the C_4_-*Panicum* at elevated as compared to ambient temperature. Although the development of CMN (particularly of the dominant *Rhizophagus* and *Funneliformis* spp.) was somewhat reduced under high temperature, plant P uptake benefits due to AMF inoculation remained well visible under both temperature regimes, though without imminent impact on plant biomass production that actually decreased due to inoculation with AMF.

## Introduction

Arbuscular mycorrhizal fungi (subphylum Glomeromycotina; Spatafora et al., 2016) are obligate symbionts of a large majority (>60%) of land plant species (van der Heijden et al., 2015). AMF play a crucial role in nutrient uptake of the host plants (Smith and Read, 2008) as well as in improving their resistance to pathogens (Newsham et al., 1995; Vigo et al., 2000) and/or tolerance to drought and osmotic stresses (Aroca et al., 2007, Augé et al., 2014, 2015). Although efficient P transfer from the soil to the host plant mediated by AMF hyphae is frequently considered to be the major benefit of AM symbiosis for their host plants (Smith and Read, 2008; Smith et al., 2011), plant uptake of N via AMF mycelium also has been demonstrated (Bücking and Kafle, 2015 and references therein). Mycorrhiza-mediated N acquisition is particularly important when soil N is predominantly in organic forms or under moderate drought (Tobar et al., 1994; Hodge and Storer, 2015; Bukovská et al., 2018 and the references therein). In return for the supply of such mineral nutrients as P or N, the host plant provides the AMF with reduced C in the forms of simple carbohydrates and/or fatty acids (Lekberg et al., 2010; Roth and Paszkowski, 2017; Řezáčová et al., 2017a). The plant C allocation into the AMF hyphae ranges between 0.9 and 20% of its gross photosynthetic production (Jakobsen and Rosendahl, 1990; Bryla and Eissenstat, 2005; Konvalinková et al., 2017; Řezáčová et al., 2017a,b; Slavíková et al., 2017).

There is only a limited host specificity in AM symbioses. The same AMF species often colonizes several plant species simultaneously and, at the same time, roots of a single plant typically host multiple AMF species (Helgason et al., 2002; Vandenkoornhuyse et al., 2003). Thus, the shared AMF mycelium often interconnects two or more plant individuals of the same or different species, thereby establishing so-called CMNs. These CMN transport nutrients and C through soil ecosystems and redistribute symbiotic benefits and costs within a plant community (Walder and van der Heijden, 2015). CMN thus affect the survival, fitness, and competitiveness of their hosts, regulate plant coexistence (van der Heijden et al., 1998; Bever et al., 2010), and maintain plant community diversity (van der Heijden et al., 1998) and ecosystem stability. It has been documented that the rates of exchange of nutrients and C between the host plants and their fungal symbionts are variable depending on partner identity, age, AMF community composition (Jansa et al., 2008; Wagg et al., 2011), C supply of the neighboring plants interconnected through the CMN (Walder et al., 2012), and/or environmental conditions (e.g., Lekberg et al., 2013). There is accumulating experimental evidence that the CMN distribute nutrients among plant partners according to their C supply rates (Lekberg et al., 2010; Kiers et al., 2011). Similarly, a plant may distribute its C among fungal partners according to their mineral nutrient supply rates (Bever et al., 2009; Walder and van der Heijden, 2015). However, the supposed reciprocal control of the C-for-nutrients exchange whereby the most beneficial partner receives the most resources (Kiers et al., 2011) may not be valid under all circumstances (Walder and van der Heijden, 2015). The partitioning of mineral nutrients among neighboring plants acquired via CMN and the associated C costs are also likely to be influenced by both plant competition and facilitation (Weremijewicz et al., 2016). Nonetheless, the exact molecular mechanisms controlling the flows of nutrients and C through CMN are not yet precisely known (Bücking et al., 2016).

Global air temperatures are expected to rise in the years and decades to come (Latef et al., 2016) and the concomitant soil warming is expected to affect organisms living in soil, including AMF that interact with plants (Compant et al., 2010). Although the average expected temperature increase is not yet very high, it may be very significant in some regions as global warming shifts the temperature/precipitation distribution patterns across continents and also as hot and cold spells become more and less likely, respectively (Lobell and Gourdji, 2012). The crucial roles of AMF in soil C cycling, ecosystem primary productivity, soil C storage or soil C losses through decomposition, while exhibiting relatively low diversity within ecosystems (Mohan et al., 2014; Treseder, 2016), show that it is necessary to understand the impact of increased temperature on the nutrient-for-C exchange between plants and AMF. Increased soil temperature mostly increases the rate of C transfer to CMN and the fungal C respiration, thus promoting more extensive extraradical hyphal network development (Rillig et al., 2002; Heinemeyer et al., 2006; Hawkes et al., 2008; Compant et al., 2013). Elevated temperatures also affect plant growth directly, often reducing photosynthesis and respiration (e.g., Hawkes et al., 2008; Latef et al., 2016). Nevertheless, AMF can sometimes decrease the impact of temperature stress on their host plant (Hawkes et al., 2008; Zhu et al., 2011) and, because the symbiotic function is dependent upon the response of all partners to the environmental context, adaptation of either symbiont could alter the nature of the symbiosis (Bunn et al., 2009). Adaptation of one plant to a certain stress may therefore possibly mitigate the negative impact of high temperatures on the other (unadapted) plants interconnected via the CMN through maintaining the mutualistic nature of the symbiosis.

Vascular plants underwent a number of adaptations during their evolution, and the C_4_ photosynthetic type is one of the most spectacular adaptive responses to high temperature, as well as to low CO_2_, water, and mineral nutrient availabilities (Ehleringer and Monson, 1993). While C_3_ plants provide the products of photosynthesis at lower metabolic costs under moderate temperatures, light levels, and water availabilities, C_4_ plants are more effective under high light and arid conditions (Waller and Lewis, 1979; Voznesenskaya et al., 2001). In a study utilizing C_4_-*Sorghum* and C_3_-*Linum*, Walder et al. (2012) found that the C_4_-*Sorghum* allocated large amounts of C to the CMN interconnecting the two plants compared to the C_3_-*Linum*, but the C_3_-*Linum* received a disproportionally large share of the isotopically (^32^P- and ^15^N-) labeled nutrient resources via the CMN. This imbalance in cost-benefit exchange, however, could be caused by the use of only one fungal species and thus absence of competition among different fungal species or incompatibility of the fungus with one or the other of the plant species. Wang et al. (2016), on the other hand, found that C_3_-*Glycine* invested more C into CMN than did C_4_-*Zea* when intercropped. Here, the results may also have been affected by what could be termed “cheating” by the sole fungal species used in the model experiment. Moreover, tripartite symbiosis – inoculation with AMF and rhizobia in parallel – may stand behind the stronger competitiveness of C_4_-maize in that particular experiment, because maize, unlike soybean, would not bear any C costs connected with biological N_2_ fixation. To predict the impact of elevated temperature on C and/or nutrient flows in plants interconnected by CMN, it is necessary to explore interactions of various plants through CMN constituted by more than one AMF species under various environmental conditions.

Toward that end, we conducted a C_3_–C_4_ plant co-existence (intercropping) experiment testing the role of AMF in the resource sharing and exploitation by a simplified plant community composed of congeneric C_3_- (*Panicum bisulcatum*) and C_4_- (*Panicum maximum*) grass species sharing CMN under ambient and at elevated temperatures. Further, we tested which of the hosts (the C_3_- or the C_4_-*Panicum*) bore the costs of C supply to the CMN under the different temperature regimes and whether nutritional benefits provided to the different plant hosts by the CMN changed upon altering the environmental conditions. To address these questions, we grew the plants in cultivation containers consisting of two plant compartments joined through a RFC. The rhizospheres of the two plants were thus spatially delimited from each other by a double root-penetration mesh barrier. To achieve a greater relevance to real field conditions as compared to majority of previous research on CMN, we inoculated the plants with a multispecies AMF community rather than, as previously, using a monospecific AMF inoculum (Walder et al., 2012; Weremijewicz et al., 2016). In the RFC, we supplied ^15^N-labeled clover litter to trace N transfer from this material to the plants via the AMF hyphae. We used organic N source because it offered a neat possibility to provide isotopically labeled N in a spatially discrete manner (much less prone to diffusion than, e.g., NO_3_^-^), such patches have previously been reported to be efficiently explored by AMF hyphae, and the ^15^N transfer from the organic amendments to plants could be used as a proxy of mycorrhizal nutrient benefits (Hodge and Fitter, 2010; Fellbaum et al., 2012; Bukovská et al., 2018).

We had expected that the C_4_-*Panicum* would generally cover a larger share of the C supply to the CMN compared to the C_3_-*Panicum*, because C_4_ plants generally show higher mycorrhizal responsiveness than do C_3_ plants (Hetrick et al., 1990; Hoeksema et al., 2010; Chandrasekaran et al., 2016) and C_4_ plants are often larger in size and thus stronger C sources. Further, we expected that the C_4_-*Panicum* would derive greater mycorrhizal benefits from the symbiosis compared to the C_3_-*Panicum*, be these growth promotion or P and/or N uptakes. Based on the reciprocal reward concept sensu Kiers et al. (2011) we also expected the C_4_-*Panicum* to cover a larger share of C supply to the CMN at elevated temperature than at ambient temperature. This is because the C_4_ plants are better adapted to warmer conditions than are the C_3_ plants.

## Materials and Methods

### Experimental Design

The experiment was set up in a fully factorial design with two factors: mycorrhizal inoculation (mycorrhiza inoculated, M+, or not, M-), and cultivation temperature (ambient [25°C during daytime, designated as “low t” from this point on] or high [36°C during daytime, designated as “high t”]). There were four replicate cultivation containers established per each inoculation and temperature treatment combination. Individuals of two different host plant species (C_3_ – *Panicum bisulcatum* and C_4_ – *Panicum maximum*) were planted in the opposite compartments of each of the cultivation containers (see Supplementary Figure S1) as a “mixed culture.” Thus, a total of 16 mixed-culture containers were set up at the beginning of the experiment. Later, 3 of these 16 containers had to be excluded due to poor growth of one or both of the plants, resulting finally in 13 mixed-culture containers retained for data analysis (yielding three replicates for both [M+ and M-] low t treatments and for the M- high t treatment). In addition, one monoculture container per each inoculation and temperature treatment combination was established with individuals of the same plant species in both compartments. The positions of the cultivation containers were completely randomized in the two growth chambers (high t or low t) and the positions were further re-randomized every week throughout the entire experiment.

### Plants

**FIGURE 1:**
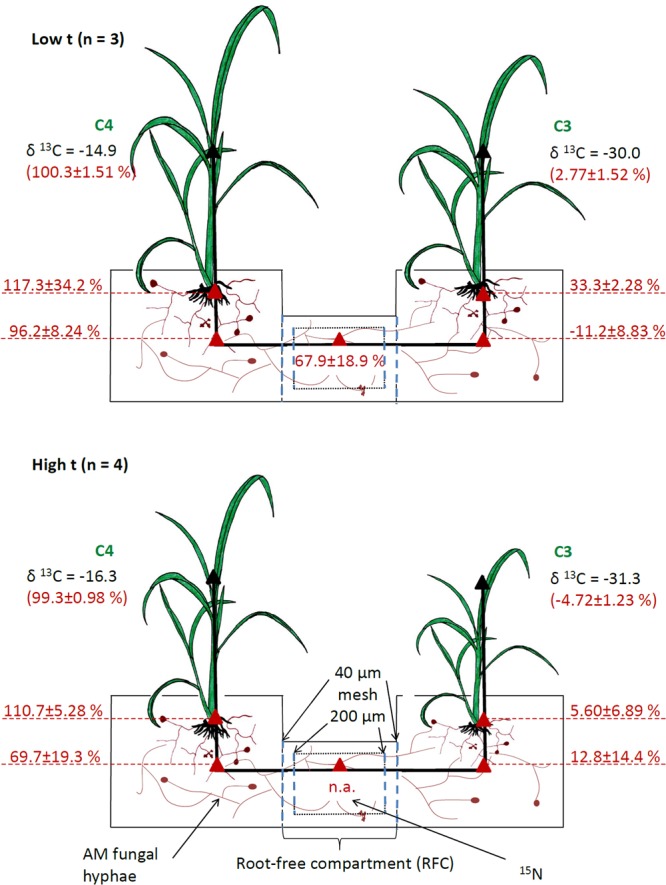
Setup of experimental containers and indications of the measured carbon source of AMF-specific fatty acid (shown in red, on a scale from 0%, representing pure C_3_-*Panicum* signature, to 100%, representing pure C_4_-*Panicum* signature derived from monoculture cultivation containers) in mixed culture containers planted with C_4_-*Panicum* on one side and C_3_-*Panicum* on the other side, and with a root-free compartment (RFC) in the middle. Mean values ± standard errors are shown separately for ambient (low t) and elevated (high t) temperature regimes. The RFC was separated from the plant compartments by root-exclusion meshes and was thus accessible only to AMF hyphae. It contained ^15^N-labeled clover biomass. The analyses were carried out using total lipid extracts from roots of both plants as well as substrate samples from both the plant and RFC compartments (red triangles). Calculations of the C source of AMF-specific fatty acid could not be carried for the RFC in the high t treatment because no significant amounts of the fatty acid were detected (n.a., not applicable). Numbers shown in black indicate mean δ^13^C values (vs. VPDB standard) measured in plant shoots of the different plant species to indicate the range of δ^13^C encountered in this research.

Two grass species belonging to the genus *Panicum* L. were used in this study: *P. bisulcatum* Thunb. and *P. maximum* Jacq. These two plant species are well characterized in terms of their photosynthesis types, with *P. bisulcatum* being a typical C_3_ plant and *P. maximum* having a C_4_ (PCK subtype) type of photosynthesis (Pinto et al., 2014). Importantly, these two plant species display different C isotopic composition characteristic of their photosynthesis types, with the C_3_ plants being significantly more ^13^C-depleted than the C_4_ plants (see **Figure 1** for data). Seeds of both plant species were kindly donated by Dr. Oula Ghannoum, Hawkesbury Institute for the Environment, University of Western Sydney, Australia. The seeds were collected from small populations of glasshouse-grown plants of the respective species, derived originally from about 20 seeds of each species collected in the fields and propagated for at least seven generations under glasshouse conditions.

### Cultivation Containers and Substrate

Plants were grown in compartmented containers consisting of two side compartments (10 cm × 10 cm × 11 cm; see **Figure 1** and Supplementary Figure S1), each planted with three plant seedlings belonging to one or the other grass species. The plants were interconnected through a middle, RFC (14 cm × 6 cm) separated from both of the plant compartments by 42 μm nylon meshes allowing AMF hyphae but not the roots to penetrate (**Figure 1**). There was a 30.5 ml labeling compartment (a tube with a diameter of 3.6 cm, 3 cm long) placed in the RFC, wrapped in a 200 μm nylon mesh on both sides (**Figure 1**), and filled with potting substrate supplemented with ^15^N-labeled white clover biomass.

The substrate consisted of thoroughly mixed (volume-based) 10% γ-irradiated (>25 kGy) field soil from Litoměřice, Czechia (N50°31′54.53″ E14°06′7.10″), 45% autoclaved zeolite MPZ 1-25 from Zeopol^1^ (grain size 1–2.5 mm), and 45% autoclaved quartz sand (grain size < 3 mm; for physicochemical properties, see Supplementary Table S1).

### ^15^N labeling

To distinguish N uptake by plants via the mycorrhizal hyphae from that taken up directly via roots, the RFC was supplemented with ^15^N-labeled biomass of white clover (*Trifolium repens* cv. Jura, Agrogen spol. s.r.o., Troubsko, Czechia) added to the labeling compartment (see above and **Figure 1**). The clover had been grown in a hydroponic tank (10 l) and fertilized with diammonium phosphate (3 g, 98% atom% ^15^N) 2 weeks after sowing, in addition to a standard Long Ashton nutrient solution including the original (1.5 mM) P concentration (Hewitt, 1966). Clover shoots were harvested 22 days after sowing, dried at 65°C for 3 days and milled to a fine powder using an MM 200 ball mill (Retsch, Haan, Germany). The clover biomass contained 38% C, 2.64% N, and 0.30% P. Isotopic enrichment of N in the labeled clover biomass reached 42 atom% ^15^N [corresponding to a δ^15^N (vs. air N_2_) = 186676]. Clover biomass was added at a rate of 1 g per 240 g of the potting substrate, mixed to homogenize and filled into the labeling compartments (40 g of the ^15^N-enriched substrate per labeling compartment). Every labeling compartment thus received with the clover biomass approximately 4.4 mg N (out of which 1.84 mg was the ^15^N), in addition to 63 mg C and 0.5 mg P. This resulted in nearly a duplication of the organic C concentration in the substrate of the labeling compartment (0.38% C as compared to 0.22% C in the rest of the cultivation containers, see also Supplementary Table S1) and in duplication of the N concentration in the substrate of the labeling compartments (0.02% N) as compared to the rest (0.01% N, see also Supplementary Table S1).

### Mycorrhizal Inoculation

Half of the containers (M+) were supplemented with 133 g mycorrhizal inoculum per each plant compartment. The inoculum consisted of potting substrate containing root fragments of leek (*Allium porrum* L.), which had been used as a host plant in a previous pot culture containing either *Rhizophagus irregularis* (N. C. Schenck and G. S. Sm.) Schüßler and Walker (2010) – Chomutov (Krak et al., 2012), *Claroideoglomus claroideum* (N. C. Schenck and G. S. Sm.) Schüßler and Walker (2010) BEG 23, or *Funneliformis mosseae* (T. H. Nicolson and Gerd.) Schüßler and Walker (2010) BEG 95. The three monospecific inocula, respectively, were mixed in the following ratio: 34 g:85 g:14 g. BEG is an abbreviation for the International Bank for the Glomeromycota^2^. Further, the mycorrhizal inoculum was supplemented with 300 spores per plant compartment of *Acaulospora tuberculata* Janos and Trappe BEG 41 and 500 spores per plant compartment of *Gigaspora margarita* W. N. Becker and I. R. Hall BEG 34 (manually picked following wet sieving and decanting). The other half of the containers (M-) received 133 g non-mycorrhizal (mock) inoculum per each of the plant compartments. The mock inoculum consisted of potting substrate containing root fragments of leek from a previous pot culture grown under the same conditions and for the same period of time as the mycorrhizal pot cultures (above) but without any AMF. The inocula were provided as layers 4–5 cm beneath the surface of the potting substrate in the plant compartments.

### Planting and Growth Conditions

Seeds of both plant species were pre-germinated in 15 cm Petri dishes on wet filter paper at 37°C for 12 h and then incubated at 35°C for 2 days, followed by incubation at 25°C for the next 2 days under ambient light. The Petri dishes with seeds were then kept in the greenhouse for another 3 days before transplanting them into the containers. Three seedlings of the respective plant species were planted in each of the plant compartments. The containers were then incubated in two identical growth chambers of the Institute of Microbiology, Prague, Czechia, providing a temperature control of ±0.5°C and light (high-pressure sodium vapor lamps combined with fluorescent tubes) intensity during the photoperiod of 300 μmol m^-2^ s^-1^ photosynthetically active radiation at plant level. For the first 26 days after planting, all plants were kept at the same growing conditions of 25/21°C average day (16 h photoperiod)/night temperatures. Thereafter, the temperatures were elevated for half of the containers to 36/32°C day/night, respectively (see Supplementary Figure S2). The containers were watered daily with deionized water to maintain approximately 85% water holding capacity of the substrate (assessed gravimetrically). From the fourth until the eighth week after planting, each plant compartment received weekly 65 ml Long Ashton mineral nutrient solution (Hewitt, 1966) with the P concentration reduced to 20% of the original recipe. This resulted in total fertilization inputs equaling 54.6 mg N and 3.0 mg P per each plant compartment over the entire duration of the experiment.

### Plant Harvest

The experimental plants were harvested 75 days after planting. The shoots were cut at the hypocotyl–root boundary and subsequently dried for 3 days at 65°C to determine the shoot dry weight. The roots were washed from the substrate under cold tap water and cut into 1 cm fragments. The roots were then divided into two parts and the fresh weights of both were recorded. One aliquot was immersed in 10% KOH (w:v) to determine the AMF colonization. The second aliquot was dried for 4 days at 65°C, weighed and the root dry weight of the whole root system was calculated as described below. Fifty grams of representative (thoroughly mixed) substrate from each of the plant compartments and the RFC were dried at 65°C for 4 days. Dried shoot, root, and substrate samples were milled to a fine powder using an MM 200 ball mill before further elemental, isotopic, and molecular analyses.

### Analyses and Calculations

Dry biomass of the roots was calculated using the fresh weight of the entire root system and dry-to-fresh biomass ratio of the roots measured on the root aliquot subjected to drying.

To determine the P concentration in plant tissues, 100 mg of a milled sample of each shoot and root were incinerated in a muffle furnace at 550°C for 12 h, the ashes were dissolved in 1 ml of concentrated (69%, w:v) HNO_3_ and briefly boiled (250°C) on a hot plate. The extracts were then transferred into volumetric flasks (50 ml) through ashless filter paper (Whatman 41, P-lab, Prague, Czechia) and made up to 50 ml with ultrapure water. Orthophosphate concentration in the extracts was measured using the malachite green method (Ohno and Zibilske, 1991). The N and C concentrations and N and C isotopic compositions in shoots, roots, and substrate samples were measured using a Flash EA 2000 elemental analyzer coupled with a Delta V Advantage isotope ratio mass spectrometer (Thermo Fisher Scientific, Waltham, MA, United States) as described earlier (Slavíková et al., 2017). Plant P and N contents were calculated from the measured nutrient concentrations in shoots and roots using the dry biomass data for the shoots and roots, respectively.

To calculate the amount of ^15^N in the plant samples which had originated from the ^15^N-labeled white clover (i.e., excess ^15^N), the measured δ^15^N values were first converted to *F*-ratios:

$$\begin{array}{c} F\;=\;(0.0036782\;\times \;(\delta /1000 + 1))/(0.0036782 \times (\delta /1000\;+\;1)\;+\;1), \end{array}$$

where δ stands for δ^15^N (‰) of the sample and 0.0036782 is the ^15^N/^14^N isotope ratio of the air.

The number of moles of N (n_N_) in the sample was then calculated as follows:

$$\begin{array}{c} n_{N}\;=\;m_{N}/(14.0030740048\;+\;{\left(15.0001088982\;-\;14.0030740048\right)}^{*}\;F), \end{array}$$

where m_N_ stands for the N content in the sample and 15.0001088982 and 14.0030740048 are the amounts (g) of 1 mol ^15^N and ^14^N, respectively.

Finally, the amounts of ^15^N in the shoots added by labeling (excess ^15^N, in moles) were calculated:

$$\begin{array}{c} {\mathrm{excess}}^{15}N\;=\;(\mathrm{Fs}\;-\;\mathrm{Fu})\;\times \;n_{N}, \end{array}$$

where Fs is the ^15^N *F*-ratio in the sample, Fu is the average ^15^N *F*-ratio in the corresponding samples of 10 unlabeled plants, and *n_N_* is the number of N moles. Here, the N isotopic composition of plants from our previous experiment established using the same substrate and the same *Panicum* plants was used to subtract the natural abundance of ^15^N.

Lipid extractions were carried out from the root and substrate samples of each of the plant compartments as well as from the RFC of each experimental container. It was performed according to the procedure described earlier by Frostegård et al. (1991) but with slight modifications: 0.1 g of dry roots or 10 g of substrate without roots was placed into a 50 ml centrifuge tube, then 15 ml of chloroform:methanol:sodium citrate buffer (0.15 M, pH 4.0) mixture (1:2:0.8, v:v: v) was added as well as 100 μl of the internal standard, nonadecanoic fatty acid (C19:0, Sigma-Aldrich, 1 μg μl^-1^ in hexane). Samples were incubated at room temperature overnight with occasional stirring, centrifuged for 10 min (750 × *g*, 16°C) and the liquid was then transferred into a new 50 ml centrifuge tube. Phase separation was achieved by adding 10 ml of the citrate buffer to each sample, intensive shaking, then centrifugation at 750 × *g* at 16°C for 2 min. The lower (organic) phase was collected with a 10 ml Hamilton syringe, filtered through a syringe-driven hydrophobic filter (Chromafil O-45/15 MS, Macherey-Nagel, Germany), then evaporated to dryness under N_2_ flow at 35°C. Fatty acids were transmethylated using the trimethylchlorosilane approach (Welc et al., 2012) and the samples were then evaporated to dryness and dissolved in hexane (100 μl). The samples were next filtered through the hydrophobic filters and analyzed using a Trace 1310 gas chromatograph (Thermo Fisher Scientific) equipped with an Rtc-5 60 m/0.25 mm ID/1 μm coating column (Restek, Bellefonte, PA, United States) coupled to a Delta V Advantage mass spectrometer via GC Isolink (Thermo Fisher Scientific). This instrumentation allowed online conversion of all fatty acids in the eluate from the GC column directly to CO_2_ and continuous measurement of the ^13^C to ^12^C ratios in the He (carrier gas)-CO_2_ flow fed subsequently to the mass spectrometer (López-Mondéjar et al., 2018).

Identification of the different fatty acids in the chromatograms was carried out by comparison with qualitative GC standards. The concentrations of the individual fatty acids in the root and substrate samples were calculated using the sample weight and quantification of the internal standard (C19:0 fatty acid) in each individual sample.

Relative contribution of the C_3_- and C_4_-*Panicum* hosts to the C contained in the AMF biomass/hyphae in the roots and in the substrate filling the different compartments of the mixed-culture containers was assessed by analyzing the isotopic composition of AMF-specific fatty acid C16:1ω5 (Olsson, 1999; Voříšková et al., 2017) in the lipids extracted from the respective root and substrate samples and comparing them to the isotopic composition of the same fatty acids extracted from the containers planted with only a single plant species. Because the amounts of C16:1ω5 fatty acid in the RFC of the M+ containers did not differ significantly from those of the M- containers at high t (Supplementary Figure S3 and Supplementary Table S2), the origin of C in the AMF biomass in the RFC was only calculated for the containers at low t (although all samples were measured, see the raw data in Supplementary Table S5). The origin of C in the C16:1ω5 fatty acid was calculated as follows:

Initially, the δ^13^C of C16:1ω5 was “cleaned off” the non-mycorrhizal background. This was done by subtraction of the isotopic contribution brought by the C16:1ω5 in the respective sample (root or substrate) from the M- containers incubated under the identical conditions (low or high t). Thereafter, the origin of C (either from the C_3_- or the C_4_-host plant) in the C16:1ω5 was derived from the reference δ^13^C values measured in the C16:1ω5 in the monoculture containers (at high t or low t, using average of two independent root samples or substrate samples from the two independent plant compartments or one RFC for each temperature regime). The contribution of the different plant species (C_3_ or C_4_) to the C detected in the C16:1ω5 was then expressed on a scale from 0% (corresponding to exclusive supply of C by the C_3_-plant) to 100% (corresponding to exclusive C supply by the C_4_-plant).

The extent of root length colonized by the AMF structures was assessed microscopically after staining the roots with Trypan blue according to Koske and Gemma (1989). We used the magnified intersection method according to McGonigle et al. (1990), scoring 100 magnified (×200) root intersections per sample.

The composition of AMF communities in the roots and in the different substrate samples was assessed on DNA extracted from the different samples using quantitative real-time PCR (qPCR) with taxon-specific markers (mt5, moss, clar, giga, and acau) targeting sequence-specific motifs of *Rhizophagus, Funneliformis, Claroideoglomus, Gigaspora*, and *Acaulospora*, respectively, in the mitochondrial (*Rhizophagus*) and nuclear (*Gigaspora, Funneliformis*, and *Claroideoglomus*) large ribosomal subunit RNA genes as described previously (Thonar et al., 2012; Couillerot et al., 2013). Abundance of *Acaulospora* in the samples from this particular experiment was quantified using a novel qPCR marker “acau” targeting the species-specific DNA motif in the nuclear large ribosomal subunit with forward primer 5′ GAG GAT TGCA GCG GAT G 3′, reverse primer 5′ CAA TCG TTA GCA AGC TAT CG 3′, and a fluorescent hydrolysis (TaqMan) probe FAM – TAG TCA CCT ACC TTC TG – BHQ1. This marker targets solely *A. tuberculata*, was designed in Allele ID version 6 software (Premier Biosoft International, Palo Alto, CA, United States), and generates an amplicon of 79 bp length. Primer annealing was set at 58°C for 30 s, in which conditions no significant cross-amplification was observed of the “acau” marker with the other AMF taxa included in our experiment, during cross-amplification testing prior to the AMF quantification in experimental samples (data not shown). Absence of cross-amplification of non-target AMF taxa included in this experiment with all the others taxon-specific markers have also been experimentally tested prior to the experiment (data not shown). The AMF taxa abundance data generated by the qPCR have been corrected for unspecific DNA losses using internal DNA standard spiked in each sample prior to DNA extraction, as described previously (Thonar et al., 2012). Solis Biodyne chemistry (5× HOT FIREPol Probe qPCR Mix Plus [ROX] master mix) was used for all qPCR assays according to manufacturer’s recommendations in a StepOnePlus^TM^ Real-Time PCR thermocycler (Applied Biosystems).

### Statistical Analyses

Analysis of variance with *p* < 0.05 as the significance cutoff level were calculated in the R 3.2.1 statistical environment (R Core Team, 2013^3^) after checking for data conformity with ANOVA assumptions (i.e., normality and homogeneity of variances). The percentage data describing the AMF colonization in the roots (assessed by microscopy) were arcsine (square-root)-transformed before the analyses. Two-way ANOVAs with mycorrhizal inoculation and cultivation temperature as factors were performed on data summed per cultivation container (i.e., total plant biomass, plant P and N contents, ^15^N excess in plant biomass, as well as the C16:1ω5 contents), and on the C_3_ share (i.e., fraction of the total assignable to the C_3_ plant contribution) in the per-container summed values of the total plant biomass, plant P and N contents, and the ^15^N excess in plant biomass. Two-way ANOVA was also performed on C16:1ω5 contents in the RFC. Further, two-way ANOVA was carried out to separate effect of the plant photosynthesis type and the temperature regimes on the data describing the C source of C16:1ω5 fatty acid in the roots and in the plant compartments of the experimental containers. One-way ANOVAs with cultivation temperature as a factor were performed on microscopically recorded levels of mycorrhizal colonization of either C_3_ or C_4_ plants, and the AMF taxon abundances (measured by qPCR) as affected by temperature (separately for the C_3_- and the C_4_-plant compartments or for the RCF). For the qPCR data, levels of mycorrhizal colonization of roots, as well as data on C source of the C16:1ω5 fatty acid, only data were used from the M+ cultivation containers. This was because the M- plants showed no signs whatsoever of mycorrhizal colonization of roots and, regarding the qPCR data, only a single M- plant root sample showed a very low abundance of *Rhizophagus* (see Supplementary Table S5 for data). Two sample *t*-tests comparisons or one-way ANOVAs with least significance difference *post hoc* tests were performed to separate means of individual factor combinations to interpret significant interactions. Two-sample *t*-test comparisons and Wilcoxon tests were carried out to test the significances of differences in the C source of the C16:1ω5 fatty acid in the different compartments (i.e., roots, substrate from rooted compartment, and the RFC) of our experimental system. Mean values and standard errors per treatment combination were calculated and are presented here in the text and/or in the figures.

## Results

### Whole Cultivation Container Level (i.e., C_3_- and C_4_-Plant Sides Summed Together)

**FIGURE 2:**
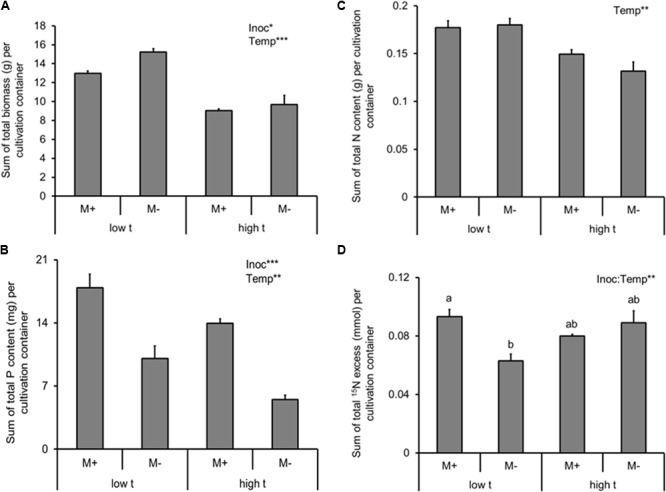
Dry biomass **(A)**, phosphorus (P) content **(B)**, nitrogen (N) content **(C)** of the experimental plants (shoot and roots combined) and ^15^N excess (i.e., ^15^N derived from the isotopically labeled clover biomass added to the RFC), **(D)** detected in the plant biomass (shoot and roots combined), summed together for cultivation containers planted with both of the *Panicum* species (mixed cultures) as affected by mycorrhizal inoculation (M+, mycorrhizal inoculum added; M-, non-mycorrhizal control) and cultivation temperature (low t, 25°C during daytime; high t, 36°C during daytime). Mean values ± standard errors are shown (*n* = 4 for M+ high t treatment, *n* = 3 for all the other treatments). Significances of inoculation (Inoc), cultivation temperature (Temp), and their interaction are indicated (factors with *p* ≥ 0.05 are not shown, ^∗∗∗^*p* < 0.001, ^∗∗^0.001 ≤*p* < 0.01, ^∗^0.01 ≤*p* < 0.05). Different letters at the individual bars indicate significant differences between the means at *p* < 0.05.

Plant biomass production (i.e., the sum of both C_3_ and C_4_ plant biomass in the same cultivation container) was systematically suppressed by both AMF inoculation as well as by elevated temperature (Supplementary Table S2 and **Figure 2A**). These effects were independent, as no significant interaction between the two factors was recorded (Supplementary Table S2).

In contrast to the biomass production, AMF inoculation strongly promoted P uptake of the plants (i.e., sum of P content of the C_3_- and the C_4_-plants growing side by side in the same cultivation container; Supplementary Table S2 and **Figure 2B**). P uptake of the model plant communities was suppressed by elevated temperature (Supplementary Table S2 and **Figure 2B**), and no significant interaction was recorded between the two factors (Supplementary Table S2).

Arbuscular mycorrhizal fungi inoculation (alone) had no significant effect on plant N acquisition (N content or excess ^15^N; Supplementary Table S2 and **Figures 2C,D**), but elevated temperature decreased N uptake by the plants (summed together for both plant compartments of each cultivation container; Supplementary Table S2 and **Figure 2C**). Elevated temperature alone had, on the other hand, no significant effect on the excess ^15^N in the plant biomass (i.e., the amount of N derived from organic fertilizer supplied to the RFC; Supplementary Table S2 and **Figure 2D**). Yet there was a significant interaction between AMF inoculation and temperature with respect to excess ^15^N (Supplementary Table S2 and **Figure 2D**), with the AMF inoculation positively affecting the ^15^N uptake by the plants at low t but having no effect at high t; **Figure 2D**).

**FIGURE 3:**
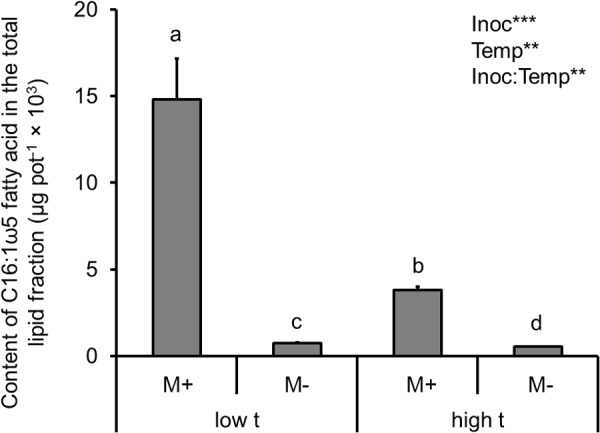
Content of the AMF-specific fatty acid (C16:1ω5) in the total lipids extracted from the root and substrate samples, summed together per cultivation containers planted with both of the *Panicum* species (i.e., roots of both C_3_- and C_4_-*Panicum*, substrate in both of the plant compartments and the RFC), as affected by mycorrhizal inoculation (M+, mycorrhizal inoculum added; M-, non-mycorrhizal control) and cultivation temperature (low t, 25°C during daytime; high t, 36°C during daytime). Mean values ± standard errors are shown (*n* = 4 for M+ high t treatment, *n* = 3 for all the other treatments). Significances of inoculation (Inoc), cultivation temperature (Temp), and their interaction are indicated (factors with *p* ≥ 0.05 are not shown, ^∗∗∗^*p* < 0.001, ^∗∗^0.001 ≤ *p* < 0.01). Bars with different letters above mean significant differences at *p* = 0.05. Different letters at the individual bars indicate significant differences between the means at *p* < 0.05.

When analyzing the effects of temperature and AMF inoculation on the total mass of AMF (as per the C16:1ω5 fatty acid content in all belowground compartments, i.e., roots in both of the plant compartments as well as in the substrate filling both of the plant compartments and the RFC), there was a strong effect recorded of both main factors (Supplementary Table S2 and **Figure 3**). Specifically, AMF inoculation strongly elevated the content of the C16:1ω5 fatty acid in the entire cultivation container as compared to the M- treatment, whereas temperature caused almost a three-fold decrease of the fatty acid content in the M+ containers at high t as compared to the M+ containers at low t (**Figure 3**). Only a small, though significant difference was recorded in the C16:1ω5 fatty acid content in the entire container between the M- treatments at low t and high t (**Figure 3**).

### Competition for Resources Between the C_3_- and the C_4_-Plants

To address the competition between the two co-existing plant species, we calculated the fraction of plant biomass, P and N yields, as well as the fraction of excess ^15^N per cultivation container assignable to the C_3_ plant (i.e., the share of resources diverted to the C_3_ plant on a whole cultivation container basis, with the remaining part of the particular resource being assignable to the C_4_ plant).

**FIGURE 4:**
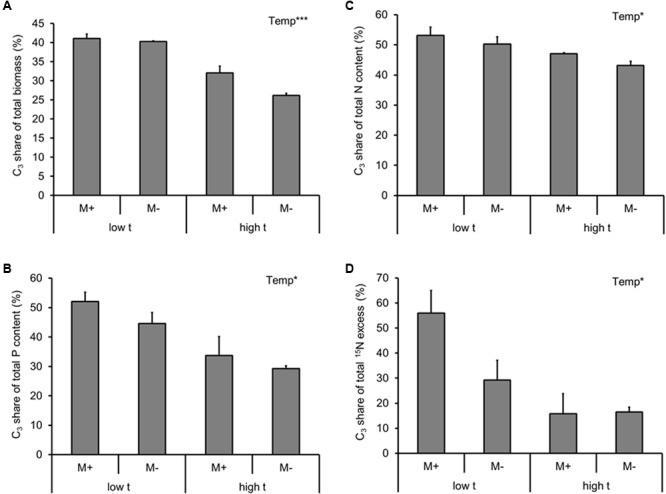
Fraction of dry biomass **(A)**, phosphorus (P) content **(B)**, nitrogen (N) content **(C)** of the experimental plants (shoot and roots combined), and the ^15^N excess (i.e., ^15^N derived from the isotopically labeled clover biomass added to the RFC), **(D)** detected in the plant biomass (shoot and roots combined) per cultivation container assignable to the C_3_ plant (i.e., the share of resources diverted to the C_3_ plant on a whole cultivation container basis, with the remaining part of the particular resource being assignable to the C_4_ species) as affected by mycorrhizal inoculation (M+, mycorrhizal inoculum added; M-, non-mycorrhizal control) and cultivation temperature (low t, 25°C during daytime; high t, 36°C during daytime) for the mixed cultures. Mean values ± standard errors are shown (*n* = 4 for M+ high t treatment, *n* = 3 for all the other treatments). Significances of inoculation, cultivation temperature (Temp), and their interaction are indicated (factors with *p* ≥ 0.05 are not shown, ^∗∗∗^*p* < 0.001, ^∗^0.01 ≤*p* < 0.05).

Based on these analyses, the C_3_ plant always lost out in the competition for resources with the C_4_ plant at high t (be it with respect to the biomass production, P uptake, N uptake, or excess ^15^N; Supplementary Table S2 and **Figures 4A–C**). For none of the measured variables there was any effect of AMF inoculation (alone or in interaction with the temperature regime; Supplementary Table S2).

### AMF Development

**FIGURE 5:**
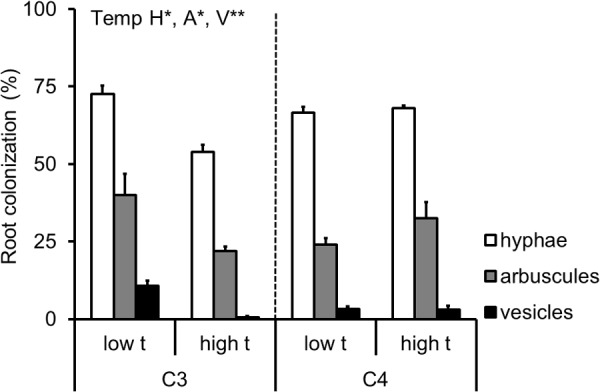
Extent of root length colonized by the mycorrhizal fungal structures of the AMF-inoculated C_3_- and C_4_-*Panicum* grasses growing side by side in the mixed culture containers. Colonization levels are shown by the hyphae (H), arbuscules (A) and vesicles (V) as assessed microscopically following staining with Trypan blue, separately for the two grass species as affected by cultivation temperature (low t, 25°C during daytime; high t, 36°C during daytime). Mean values ± standard errors are shown (*n* = 4 for high t treatment and *n* = 3 for the low t treatment). Significance of temperature treatment is indicated for the C_3_ plants only (^∗^0.05 > *p* ≥ 0.01, ^∗∗^0.01 > *p* ≥ 0.001) because there was no statistically significant effect of temperature recorded for any of the parameters in the C_4_ plants.

Roots of all M+ plants were highly colonized by the AMF, and the roots of M- plants remained free of AMF structures throughout the entire experiment. On average (± standard error), the hyphae, arbuscules, and vesicles occupied 65 ± 2%, 29 ± 3%, and 4 ± 1% of the root length of the M+ plants, respectively. The extent of root colonization of the C_3_-*Panicum* was strongly reduced under elevated temperature, whereas no effect of temperature was recorded on the root colonization levels of the C_4_-*Panicum* (Supplementary Table S3 and **Figure 5**).

**FIGURE 6:**
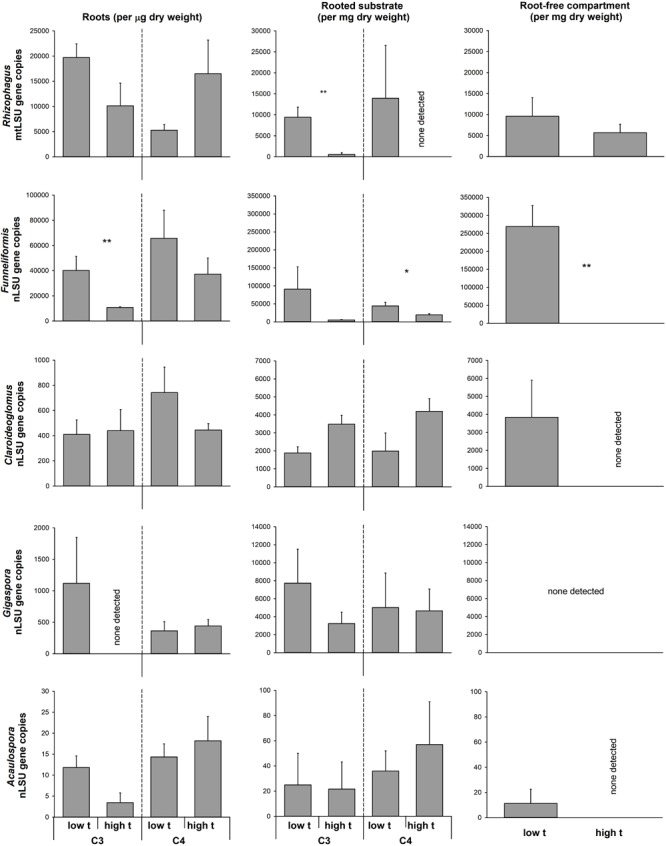
Abundance of the different arbuscular mycorrhizal fungal taxa in the roots and substrate samples from the plant- and the RFCs of the mycorrhiza-inoculated cultivation containers containing both C_3_ and C_4_ plant species, as assessed by quantitative real-time PCR with taxon-specific markers for mitochondrial or nuclear large ribosomal subunit (mtLSU or nLSU, respectively). Statistically significant differences are shown between the different temperature regimes (low t, 25°C during daytime; high t, 36°C during daytime), separately for the C_3_ and for the C_4_ plants, if applicable. Mean values ± standard errors are shown (*n* = 4 for high t treatment and *n* = 3 for the low t treatment). ^∗^0.05 > *p* ≥ 0.01, ^∗∗^0.01 > *p* ≥ 0.001.

Although the data on individual AMF taxa abundance in roots and in the potting substrate generated by the qPCR were quite noisy, there were a few cases of a significant reduction of abundance of *Rhizophagus* and *Funneliformis* (the two dominant taxa in our experiment) due to elevated temperature (**Figure 6**). No significant stimulation was observed of abundance of any of the AMF taxa in any of the experimental compartments by elevated temperature (**Figure 6**). Importantly, the abundance of none of the AMF taxa in C_4_-plant roots was reduced by elevated temperature, whereas abundance of *Funneliformis* was significantly reduced upon elevated temperature in the C_3_-plant roots as compared to ambient temperature (**Figure 6**). *Funneliformis* appeared to be most susceptible to elevated temperature in our experiment, whereas *Claroideoglomus, Gigaspora*, and *Acaulospora* (all three being not particularly abundant in our samples, though) did not indicate any change in abundance in any of the measured compartments due to the elevated temperature (**Figure 6**).

### The C Source of CMN Interconnecting the C_3_ and C_4_ Plants

Elevated temperature alone did not affect the isotopic composition of C in the AMF-specific fatty acid in either roots or substrate from the plant compartments, whereas clear effect could be assigned to the photosynthesis type of the plant (C_3_ vs. C_4_) growing in the respective compartment (Supplementary Table S4). The data were thus pooled across both of the temperature regimes for all further statistical analyses (but displayed separately for low t and high t in **Figure 1**). Due to the constraints detailed above (i.e., no significant amounts of C16:1ω5 fatty acid detected in the RFC of the M+ containers, Supplementary Figure S3), any meaningful calculations could only be made for the RFC from the containers incubated at low t (for raw data, see Supplementary Table S5). Carbon isotopic composition of the C16:1ω5 fatty acid in the C_3_-*Panicum* roots carried a clear C_3_ imprint (on average across both temperature regimes 17.5 ± 6.2%, with 0% representing a pure C_3_-isotopic signature), which was not significantly different (*p* = 0.2, *t*-test) from the isotopic imprint within the C16:1ω5 fatty acid in the substrate of the C_3_ plant compartment (2.5 ± 9.0% across both temperature regimes). For numbers for each of the temperature regimes separately, see **Figure 1**, for raw data, see Supplementary Table S5.

Similarly, the C isotopic composition of the C16:1ω5 fatty acid in the roots and in the substrate of the C_4_ plant compartments both carried a clear C_4_-isotopic imprint (113.6 ± 12.3% and 81.0 ± 29.5%, respectively, **Figure 1**) and did not differ significantly from each other (*p* = 0.1). There was a significant difference in the C isotopic imprint within the C16:1ω5 fatty acid recorded in the RFC (at low t) and the substrate of the C_3_ plant compartment analyzed at both high t and low t (*p* < 0.01), whereas no significant difference was recorded between the substrate beneath the C_4_ plants (at both temperature regimes) and the RFC at low t (*p* = 0.6). The difference in C isotopic imprint of the C16:1ω5 fatty acid between the two plant compartments (C_3_ vs. C_4_) was highly significant (*p* < 0.001), and so it was between the roots of the C_3_ and C_4_ plants (*p* < 0.001).

## Discussion

### Mycorrhizal Effects on Plant Biomass and Nutrient Uptake at Different Temperatures

Mycorrhizal inoculation strongly increased P content of the plants (nearly two-fold at low t and even more so under high t, **Figure 2B**), which confirmed that the symbiosis was fully functional under both temperature regimes in terms of P uptake improvement of the plants. Bit surprisingly though (but see also Řezáčová et al., 2017b, 2018), establishment of mycorrhizal symbiosis did not increase biomass production of our experimental plants (actually it did decrease it by about 10–15%, **Figure 2A**), most likely because of C limitation, due to temperature stress (of which the latter was, however, not measured directly, e.g., by assessing molecular stress markers or efficiency of photosynthetic C fixation) or other constraints (see also Johnson et al., 2015 for further discussion). N limitation of growth of the M+ plants due to competition for N between the plants and the AMF and/or their associated bacteria (such as described, e.g., by Saia et al., 2014 or Püschel et al., 2016) is not a likely explanation of the observed growth reduction because the N content of the plants was actually not affected at all by mycorrhiza formation (**Figure 2C**).

Elevated temperature strongly and negatively affected plant biomass, P and N contents of the mixed plant stands (**Figure 2**, most notable was the reduction of M- plant P content by more than 50% due to elevated temperature), eventually resulting in a significant suppression of the C_3_ plant growth and nutrient uptake in the mixed culture containers (**Figure 4**). Yet there was no significant interaction between the factors of temperature and inoculation for any of the above parameters, indicating that mycorrhiza did not increase to any significant degree the tolerance (in terms of plant biomass production or mineral nutrition) of their host plants to elevated temperature. This result was quite surprising given other reports showing increased tolerance of the host plants to extreme temperatures due to formation of mycorrhizal symbiosis (e.g., Bunn et al., 2009).

The biggest surprise was certainly the pattern of plant uptake of ^15^N from the RFC (**Figure 2D**). Whereas M+ plants obtained significantly more ^15^N than the M- plants from the organic N source at low t (and nearly significantly, *p* = 0.07, it was the C_3_ plant that was preferentially served by the mycorrhizal networks with the ^15^N at low t, as compared to M- conditions, **Figure 4D**), there was no significant difference between the M+ and M- plants at high t (**Figure 2D**). These results indicate that the decomposition of organic N amendment followed different trajectories at the different temperatures and that mycorrhiza was an important shaper of the decomposition and/or plant N uptake (consistent with earlier and recent reports by, e.g., Leigh et al., 2009; Hodge and Fitter, 2010; Bukovská et al., 2018) from the organic N source at low t only. At high t, in contrast, it seems that plant uptake of ^15^N from the organic N source was mostly dominated by passive diffusion of inorganic N species toward the roots rather than N uptake via the mycorrhizal pathway (**Figure 2D**). This notion is also consistent with the lower abundance of AMF biomass in the RFC at high t as compared to low t (be it based on the AMF-signature fatty acid analyses, **Figure 3**, or by means of the qPCR analyses, **Figure 6**). It also seems that N decomposition was faster at high t (consistent with a report by Dessureault-Rompré et al., 2010 and references therein), resulting in nearly equal transfer of ^15^N (in terms of its mass) to the plants at both temperature, whereas total N uptake actually decreased in response to elevated temperature (**Figure 2**).

### Who Was Feeding the CMN at the Different Temperatures?

Based on the facts that C_3_ and C_4_ plants have very distinct isotopic composition (δ^13^C values) of C in their biomass (**Figure 1**) and that these differences extend to the AMF hyphae associated with such host plants (Querejeta et al., 2003; Courty et al., 2015), we could disentangle the C source of the AMF hyphae in our experiment. Specifically, we could address the question, which of the plant species was preferentially feeding the CMN under the different temperature regimes. To this end, we employed stable isotopic analyses of the AMF-signature fatty acid (C16:1ω5) both in plant roots and in the potting substrate of the different compartments of our experimental containers populated with the same or different plant species. This concept had recently also been described in detail by Walder et al. (2013).

To our surprise, we were not able to obtain evidence for preferential feeding of the shared mycorrhizal network by the C_4_ plant (at least under high t). Instead, the data showed that there were actually no changes in the pattern of feeding the CMN by one or the other plant species with increasing temperature, nor there was any overall asymmetry in feeding the CMN by the two different plant hosts. Our results thus demonstrate a very balanced investment of the two plants into the CMN and that this investment is not substantially affected by elevated temperature. Our data are thus in direct contrast to previous research showing that one or the other plant dominates the C supply to the CMN (Walder et al., 2012; Wang et al., 2016). The previous experiments had all used phylogenetically more distant plant species pairs, and it has been shown that plant size and/or presence of rhizobia could affect the C inputs into the CMN (Nakano et al., 1999; Wang et al., 2016; Weremijewicz et al., 2016). Because we used plant species closely related phylogenetically and of similar size and growth rates (although not necessarily so under all temperatures), such asymmetry in C feeding of the CMN as seen in previous experiments may not have developed in our case. Further, because for the first time to our knowledge in an experiment addressing C supply to CMN we used a synthetic community of the AMF instead of a single AMF isolate/genotype, the two different plant species could have preferentially associated with different AMF taxa, doing so with unknown consequences for the formation of the CMN. Nonetheless, molecular community analyses indicated that the plants did indeed share the same AMF taxa to a great extent (**Figure 6** and Supplementary Table S5). Further, both *Rhizophagus* and *Funneliformis*, which actually formed dominants of our realized AMF communities (**Figure 6**), are clearly capable of crossing the distances of several centimeters between the two root compartments (Jansa et al., 2005). Somewhat confounding, however, was the relatively low abundance of these dominant AMF taxa in the RFC at high t (particularly surprising was the apparent absence of *Funneliformis* there, **Figure 6**) and the concomitant low detection of the AMF-specific fatty acid in the RFC at high t. This might indicate rather low colonization of the RCF at high t by the AMF hyphae, provided the C16:1ω5 is a good AMF biomass marker at all temperatures (which may not in fact be the case [cf. Sumner et al., 1969]). What would be needed here is a direct proof of functional hyphal interconnection (or lack of it) between the two plants, which is currently not easy to provide. A further confounding factor may be that we actually used the substrate collected from the labeling compartment as a proxy for the entire RFC to assess the AMF communities there both by the fatty acid and the qPCR approaches. The labeling compartment was enriched with organic N, but there was still some organic N-unenriched volume in the RFC (**Figure 1**), which we did not sample, however. Uncontrolled development of high t-specific microbial communities is at least theoretically possible in organic N-enriched patches (though the composition of saprotrophic microbes was not experimentally assessed here). And because not only synergistic, but also antagonistic interactions between certain soil microbes and AMF hyphae are known (e.g., Svenningsen et al., 2018), the observed suppression of some, though not all, AMF (**Figure 6**) in the RFC should be interpreted cautiously with respect to the CMN formation between the two side compartments of the cultivation containers.

### Mycorrhizal Communities in and Outsides of Roots at the Different Temperatures

Interestingly, we observed a significant decrease in AMF colonization rates (as assessed microscopically) at high t as compared to low t in the roots of the C_3_-*Panicum* (**Figure 5**). Because such a decrease was not observed in the C_4_-*Panicum*, it seems that the AMF *per se* (at least those in the roots) were not particularly sensitive to elevated temperatures (probably with the only exception of *Funneliformis* in our experiment, **Figure 6**). This would be consistent with previous observations by others, e.g., Bunn et al. (2009), showing little sensitivity of both high temperature-adapted and -unadapted AMF genotypes to elevated temperatures. Further, these results indicate that the C_3_-*Panicum* actively suppressed the development of the (dispensable) root symbionts (particularly *Funneliformis*, see **Figure 6**) at high t, where the plants were obviously severely stressed by the elevated temperature and/or presence of the C_4_ neighbor (**Figure 4**). On the other hand, it remains to be explained why the root colonization by all of the AMF taxa was retained in the C_4_ plants exposed to high t at levels comparable to those under the low t conditions. Most likely the C_4_ plants, because of their better adaptation to elevated temperatures, were better able to continue benefitting (nutritionally) from the symbiosis at high t than the C_3_-*Panicum* and thus they also returned larger amounts of their C back to their symbionts to maintain the high root colonization levels (Kiers et al., 2011).

In the substrate beneath the plants and particularly in the RFC, suppression of both *Funneliformis* and (occasionally) also of *Rhizophagus* (**Figure 6**) at high t was observed as compared to ambient temperature. This is interesting because these two AMF genera often dominate field AMF communities, particularly in the cropped soils (e.g., Jansa et al., 2014; Davison et al., 2015) and can confer great nutritional benefits to their host plants (Jansa et al., 2005, 2008). Reduction of their hyphal development at high t would possibly explain the lower amounts of AMF-specific fatty acid at high t detected in the experimental containers as compared to low t (**Figure 3**). We know from another experiment carried under ambient temperature that at least *Rhizophagus* hyphal development should be stimulated by the clover biomass added to the RFC (Bukovská et al., 2018). Indeed, both of the dominant AMF (*Funneliformis* and *Rhizophagus*) and at least two of the subdominants (*Claroideoglomus* and *Acaulospora*) developed decently in the RFC at low t, whereas *Gigaspora* was possibly not able to grow through the root-exclusion meshes (consistent with previous reports by Smith et al., 2004). Why the AMF except *Rhizophagus* actually disappeared from the RFC at high t (qPCR detection limit permitting, Thonar et al., 2012) remains unclear – but see discussion on the possible antagonistic biotic interactions above. It should also be tested in the future whether the capacity of the AMF to penetrate nylon meshes (which is generally very low for *Gigaspora* for instance) would be more restricted for the other AMF at high t than at low t (as our results suggest). It should also be tested whether hyphal development at large distance is restricted more at high t than at low t, e.g., due to larger metabolic costs of hyphal maintenance (Heinemeyer et al., 2006) or due to more severe antagonistic interactions with other soil biota (Svenningsen et al., 2018).

## Conclusion

With respect to ongoing global changes and because CMN may reportedly mitigate at least some of the stresses experienced by their host plants (Lenoir et al., 2016; Millar and Bennett, 2016), it may become increasingly important to study the widespread effect of CMN on nutrient and C flows between plants and soil in complex plant and AMF communities subjected to various environmental conditions (Fitter et al., 2000; Heinemeyer et al., 2006). Not always the AMF help withstand stress (e.g., Pollastri et al., 2018), but sometimes (like here) at least the mineral nutrition of plants is improved upon symbiosis with AMF, if not the biomass production. Although we identified a number of technical issues that need further attention such as the need of direct proof regarding efficient formation of CMN, or the stability of C16:1ω5 synthesis under different temperatures, we clearly confirmed suitability of the AMF-signature fatty acid-based approach for tracking AMF C source to address the dynamics of C gains by the CMN supported by both C_3_ and C_4_ plants (first described by Walder et al., 2012). We also showed that extraradical AMF hyphae seem to be more susceptible to high temperatures than the AMF biomass in the roots. Our *Funneliformis* isolate (one of the dominant AMF in our experiment) was particularly strongly affected by elevated temperature, seemingly more than our *Rhizophagus* isolate or the other (subdominant) AMF genera – a path of research that certainly deserves more attention in the future. Particular attention should also be dedicated in the future to better description of the rates of organic N decomposition at different temperatures and how these (directly or interactively with other factors) affect the mycorrhizal N uptake pathway and nutrient-for-C trades at the symbiotic interface (sensu Fellbaum et al., 2014). Last but not least, more C_3_–C_4_ congeneric pairs should be employed as model hosts in similarly designed experiments as ours to allow generalizations of the results to a broader range of plant taxa (see Řezáčová et al., 2018 for further discussion).

## Author Contributions

JJ, LZ, OB, DP, TK, MH, and RS planned, designed, and carried out the experiments, including the different physicochemical and molecular analyses. VŘ and JJ analyzed the data and wrote the manuscript. All authors approved the final version of the manuscript.

## Conflict of Interest Statement

The authors declare that the research was conducted in the absence of any commercial or financial relationships that could be construed as a potential conflict of interest.

## Footnotes

1 www.zeolity.cz

2 www.i-beg.eu

3 http://www.R-project.org/

## Abbreviations

AM: arbuscular mycorrhiza
AMF: arbuscular mycorrhizal fungi
ANOVA: analysis of variance
C: carbon
C16:1ω5: AMF-specific fatty acid
CMNs: common mycorrhizal networks
M-: non-mycorrhizal control
M+: mycorrhiza inoculated
N: nitrogen
P: phosphorus
qPCR: quantitative real-time PCR
RFC: root-free compartment
