# Supplementary material for: Little Cross-Feeding of the Mycorrhizal Networks Shared Between C3-Panicum bisulcatum and C4-Panicum maximum Under Different Temperature Regimes
Source: Front Plant Sci. 2018 Apr 6;9:449. doi: 10.3389/fpls.2018.00449 (PMC5897505; doi:10.3389/fpls.2018.00449)

Supplementary Material

Little cross-feeding of the mycorrhizal networks shared between C_3-*Panicum bisulcatum* and C_4-*Panicum maximum* under different temperature regimes

Veronika Řezáčová, Lenka Zemková, Olena Beskid, David Püschel, Tereza Konvalinková, Martina Hujslová, Renata Slavíková and Jan Jansa*

*** Correspondence:** * Jan Jansa: jansa@biomed.cas.cz

TABLE S1 | Physicochemical properties of potting substrate mixed from previously sterilized components (soil : zeolite : sand, 10 : 45 : 45, v: v: v) and used to fill the experimental containers as well as the root free compartment. pH (pH_{water}) was measured in aqueous soil suspension (1 : 2.5, w : v) after shaking for 1 h. Total phosphorus (P) was assessed colorimetrically following incineration of the substrate at 550°C and extraction with hot 14 M HNO_3. Water-extractable P was measured colorimetrically in aqueous soil extract (1 : 10, w : v) after shaking the suspension for 20 h and filtration through 0.2 µm membrane filter. Total nitrogen (N) and organic carbon (C) as well as isotopic composition of the C were assessed using a Flash EA 2000 elemental analyzer coupled with a Delta V Advantage isotope ratio mass spectrometer (Thermo Fisher Scientific, Waltham, MA, USA). Isotopic composition of the C is given with respect to the international Vienna Pee Dee Belemnite (VPDB) standard.

	mean value of 3 analytical reps
pH_{water}	8.90
Total P (mg kg^{-1})	46.5
Water-extractable P (mg kg^{-1})	2.95
Total N (%)	0.01
Total organic C (%)	0.22
δ^{13}C (vs. VPDB standard, in ‰)	−18.11

TABLE S2 | Effect of mycorrhizal inoculation (Inoc), cultivation temperature (Temp) and their interaction on sums per cultivation container of total dry biomass of the plants (Total DW), plant phosphorus (P) and nitrogen (N) contents, ^{15}N excess in shoot and roots, and content of AMF-specific fatty acid (C16:1ω5) in all compartments, C_3 share (i.e., the fraction of total amount assigned to the C_3 plant) of total biomass, plant P and N contents, and ^{15}N excess in shoots and roots, and the C16:1ω5 content in root free compartment (RFC). *F*-values (F) and levels of statistical significance (*p*-value ranges) as per two-way ANOVA are shown. n = 4 for high t M+ treatment and n = 3 for low t (both M+ and M−) and high t M− treatments. Asterisks indicate levels of statistical significance as follows: *** $p < 0.001$, ** $0.001 \leq p < 0.01$, * $0.01 \leq p < 0.05$, ns indicates $p \geq 0.05$.

		Inoc		Temp		Inoc:Temp	
		F	*p*-value	F	*p*-value	F	*p*-value
**Sums per cultivation container**	**Total DW**	7.95	*	58.60	***	1.81	ns
	**Total P content**	41.34	***	11.98	**	0.06	ns
	**Total N content**	0.40	ns	19.95	**	1.48	ns
	**Total ^{15}N excess**	2.62	ns	0.69	ns	10.66	**
	**C16:1ω5 content**	35.08	***	19.99	**	16.38	**
**C_3 share**	**Total DW**	3.28	ns	58.35	***	2.89	ns
	**Total P content**	0.71	ns	9.22	*	0.07	ns
	**Total N content**	1.85	ns	8.72	*	0.05	ns
	**Total ^{15}N excess**	1.28	ns	9.26	*	2.31	ns
**C16:1ω5 content in RFC**		11.10	**	13.03	**	11.16	**

TABLE S3 | Effect of cultivation temperature (Temp) on the development of arbuscular mycorrhizal fungi (AMF) assessed microscopically in roots of either C_3 or C_4 plants. *F*-values (F) are shown alongside with the levels of statistical significance (*p*-value ranges) from one-way ANOVAs. Only mycorrhiza-inoculated cultivation containers (M+) were included in these analyses. n = 4 for high t and n = 3 for low t treatment. Asterisks express levels of statistical significance as follows: ** $0.001 \leq p < 0.01$, * $0.01 \leq p < 0.05$, ns indicates $p \geq 0.05$.

**Experimental factor**		**Mycorrhizal colonization of roots (root length colonized)**					
		**C_3**			**C_4**		
		**Hyphae**	**Arbuscules**	**Vesicles**	**Hyphae**	**Arbuscules**	**Vesicles**
Temp	F	11.49	9.55	37.53	0.19	1.59	0.27
	p-value	*	*	**	ns	ns	ns

TABLE S4 | Effect of cultivation temperature (Temp), plant photosynthesis type (Plant), and interaction of those two factors, on the data describing the carbon (C) source of C16:1ω5 fatty acid in roots and in the substrate filling the plant compartments on a scale from 0% (pure C_3-signature) to 100% (pure C_4-signature). *F*-values (F) are shown alongside with the levels of statistical significance (*p*-value ranges) from two-way ANOVAs. Only mycorrhiza-inoculated cultivation containers (M+) were included in these analyses. n = 4 for high t and n = 3 for low t treatment. Asterisks express levels of statistical significance as follows: *** $p < 0.001$, ns indicates $p \geq 0.05$.

**Experimental factor**		**C source of the C16:1ω5 fatty acid**	
		**Roots**	**Substrate (plant compartments)**
Temp	F	1.27	0.01
	p-value	ns	ns
Plant	F	38.52	29.55
	p-value	***	***
Temp:Plant	F	0.48	2.80
	p-value	ns	ns

FIGURE S1 | Appearance of an experimental container and its dimensions. The container consisted of three parts. The two side compartments were planted with two different or the same *Panicum* grasses. The third (middle, root-free compartment) was separated from the two lateral compartments on both sides by 42 µm nylon meshes allowing AMF hyphae but not roots to pass through. The middle compartment contained a 30.5 ml labelling patch (a PVC tube with inner diameter of 3.6 cm and 3 cm long), separated from the surrounding substrate by 200 µm nylon mesh at both ends, and filled with the potting substrate supplemented with ^{15}N-labelled white clover biomass. Apart from the labelling patch, all compartments were filled with the unamended potting substrate described in Table S1.

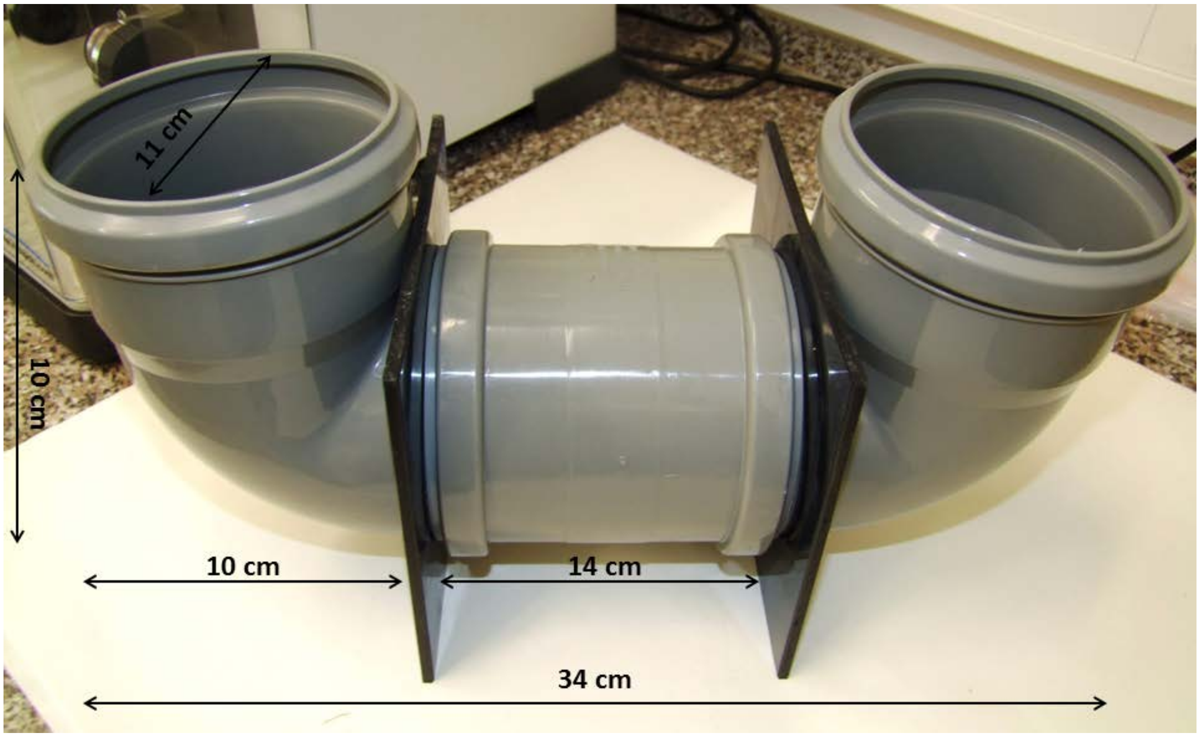

FIGURE S2 | Temperatures in two growth chambers of the Institute of Microbiology, where the experiment was conducted, as measured by two independent temperature probes connected to a data logger (Hobo H8, Onset Computer Corporation, Bourne, MA, USA). Temperatures were recorded every 15 min. All plants were kept at the same growing conditions for the first 26 days of the experiment (temperatures being 25°C and 21°C during day [16 h] and night [8 h], respectively). Thereafter, temperatures were elevated in one growth chamber (Temperature 2) to 36/32°C (day/night) while remaining unchanged in the other chamber (Temperature 1).

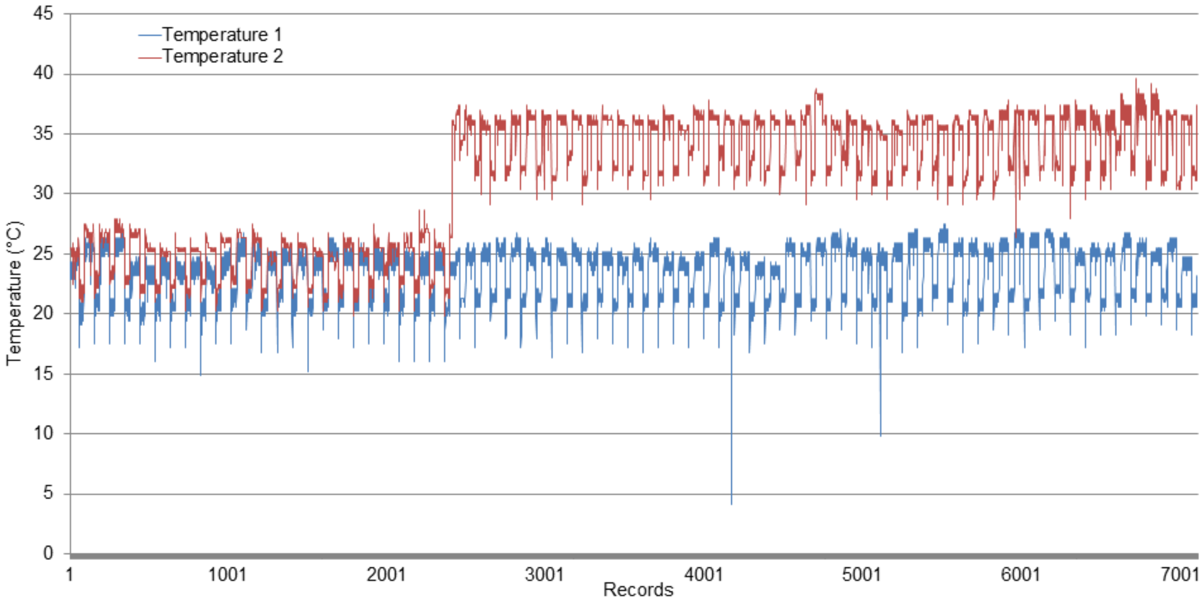

FIGURE S3 | Content of the AMF-specific fatty acid (C16:1ω5) in the total lipid extracts from substrate of the root-free compartment (RFC) as affected by mycorrhizal inoculation (M+, mycorrhizal inoculum added; M-, non-mycorrhizal control) and cultivation temperature (ambient – low t, 25°C during daytime; elevated – high t, 36°C during daytime). Mean values and standard errors are shown (n = 4 for M+ high t treatment, n = 3 for both of the low t treatments and for the M- high t treatment). Significances of inoculation (Inoc), cultivation temperature (Temp), and their interaction as per two-way ANOVA are indicated (factors with $p \geq 0.05$ are not shown, ** $0.001 \leq p < 0.01$. Different letters at the individual bars indicate significant differences between the means at $p < 0.05$.

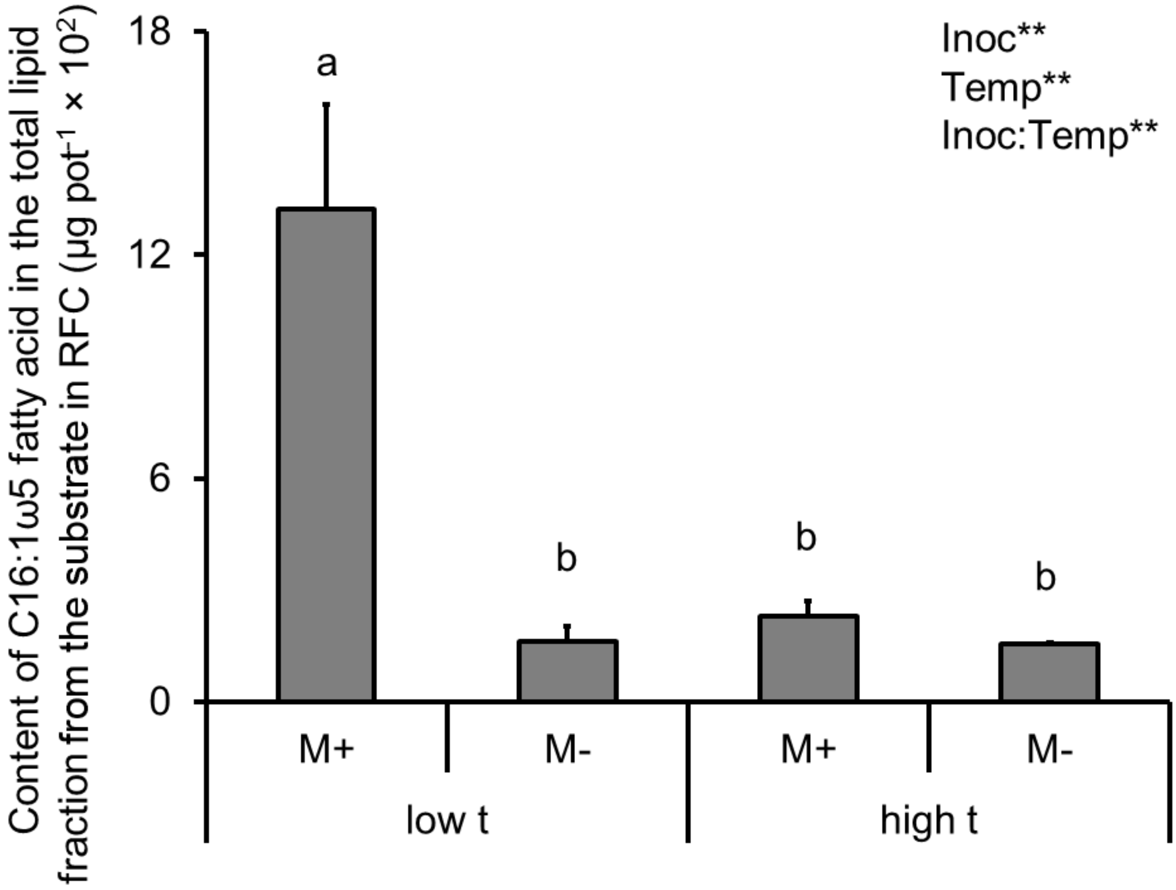

Supplement: Supplementary file 2 [file Presentation_1.PDF]
